# Bacterial SOS May Be the Key to Combating Antibiotic Resistance

**DOI:** 10.1371/journal.pbio.0030221

**Published:** 2005-05-10

The development of antibiotic drugs changed the face of clinical medicine forever, and, for a short while at least, it seemed that a perfect cure for bacterial infections had been discovered. But widespread overuse and misuse of antibiotics over the last several decades has proved the Achilles heel of this once seemingly invincible class of drugs and has fostered bacterial resistance to conventional antibiotic therapies. New drugs are continually being developed to replace those crippled by resistance, but despite scientists' best efforts, new “superbugs” are evolving faster than the drugs required to control them.

For some time now, drug resistance has been considered a consequence of errors (called mutations) that accumulate spontaneously during replication of the bacterial genome. In many cases those mutations are either inconsequential or harmful to the bacteria, but on rare occasion, they provide an accidental benefit: resistance to the drugs that kill them. Because the mutations were assumed to be spontaneous, there was no obvious way to prevent them and thus antibiotic resistance appeared inevitable. But some researchers are taking a pro-active approach by testing the assumptions about how mutations are made. For one group of bacterial researchers, this approach may have paid off.

Chemists at the Scripps Research Institute and the University of Wisconsin have uncovered evidence that spontaneous mutations are not the only way in which bacteria acquire resistance to antibiotics. It appears that the bacteria, rather than passively waiting around for a lucky break, may play an active role in their own evolution. The key is in the way antibiotics interact with their bacterial targets. Quinolone antibiotics are a relatively new class of antibiotics that work by interfering with proteins called topoisomerases, which assist DNA replication by loosening tightly wound DNA and making it accessible. In order to do this, the topoisomerase must break the DNA strands and fill in the gap with a temporary protein bridge. Under normal circumstances, the bridge is removed and the DNA is reconnected after the topoisomerase has done its job, but quinolones bind to this protein bridge and prevent the DNA from resealing. The freed double-strand ends signal that DNA damage has occurred and activate the cell's repair pathway.

According to Ryan Cirz et al., DNA damage, induced by antibiotics or other stressors, sets off a bacterium's emergency repair mechanism: the SOS DNA damage response. Under normal conditions, the genes are turned off by a special repressor protein called LexA. In response to the damaged DNA, the LexA repressor is cleaved and no longer inhibits transcription of the SOS response genes. Cirz et al. propose that antibiotic-mediated DNA damage generates a reduction in the concentration of LexA that is sufficient to increase the expression of three nonessential DNA polymerases shown to be required for mutation: Pol II (encoded by the gene *polB*), Pol IV (encoded by *dinB*), and Pol V(encoded by *umuD* and *umuC*). Together these polymerases promote DNA repair—and cause mutations in bacterial DNA that can lead to antibiotic resistance.

This suggests that quinolone antibiotics (and other antibiotics that cause similar kinds of DNA damage) may increase the likelihood that bacteria will evolve resistance and that new generations of drugs will have little chance of succeeding where today's drugs have failed. But all hope is not lost. After showing that the evolution of quinolone resistance depends on activating the SOS response genes gated by LexA, Cirz et al. go on to demonstrate that blocking LexA cleavage, in vitro and in a mouse model, prevents mutation and results in bacteria that are unable to evolve antibiotic resistance. Thus, developing novel therapeutic agents that target LexA or the associated SOS pathway may prove a promising strategy for controlling the spread of the superbugs.[Fig pbio-0030221-g001]

**Figure pbio-0030221-g001:**
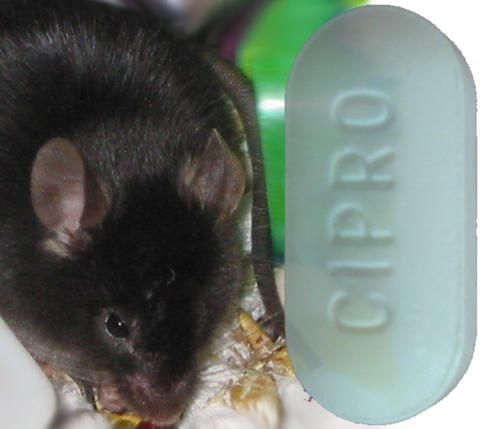
Inhibiting a protein involved in DNA repair in bacteria might prevent mutations that promote antibiotic resistance, prolonging the effectiveness of ciprofloxacin and other quinolone antibiotics

